# Mitochondrially targeted vitamin E succinate efficiently kills breast tumour-initiating cells in a complex II-dependent manner

**DOI:** 10.1186/s12885-015-1394-7

**Published:** 2015-05-13

**Authors:** Bing Yan, Marina Stantic, Renata Zobalova, Ayenachew Bezawork-Geleta, Michael Stapelberg, Jan Stursa, Katerina Prokopova, Lanfeng Dong, Jiri Neuzil

**Affiliations:** 1 School of Medical Science, Griffith University, Southport, Qld 4222, Australia; 2 The Department of Chemistry of Natural Compounds, University of Chemistry and Technology, Prague, Czech Republic; 3 Institute of Biotechnology, Academy of Sciences of the Czech Republic, Prague, 142 20 Czech Republic

**Keywords:** Tumour-initiating cells, Mitochondrially targeted vitamin E succinate, Complex II, Mitochondrial potential, Mitochondria, Breast cancer

## Abstract

**Background:**

Accumulating evidence suggests that breast cancer involves tumour-initiating cells (TICs), which play a role in initiation, metastasis, therapeutic resistance and relapse of the disease. Emerging drugs that target TICs are becoming a focus of contemporary research. Mitocans, a group of compounds that induce apoptosis of cancer cells by destabilising their mitochondria, are showing their potential in killing TICs. In this project, we investigated mitochondrially targeted vitamin E succinate (MitoVES), a recently developed mitocan, for its *in vitro* and *in vivo* efficacy against TICs.

**Methods:**

The mammosphere model of breast TICs was established by culturing murine NeuTL and human MCF7 cells as spheres. This model was verified by stem cell marker expression, tumour initiation capacity and chemotherapeutic resistance. Cell susceptibility to MitoVES was assessed and the cell death pathway investigated. *In vivo* efficacy was studied by grafting NeuTL TICs to form syngeneic tumours.

**Results:**

Mammospheres derived from NeuTL and MCF7 breast cancer cells were enriched in the level of stemness, and the sphere cells featured altered mitochondrial function. Sphere cultures were resistant to several established anti-cancer agents while they were susceptible to MitoVES. Killing of mammospheres was suppressed when the mitochondrial complex II, the molecular target of MitoVES, was knocked down. Importantly, MitoVES inhibited progression of syngeneic HER2^high^ tumours derived from breast TICs by inducing apoptosis in tumour cells.

**Conclusions:**

These results demonstrate that using mammospheres, a plausible model for studying TICs, drugs that target mitochondria efficiently kill breast tumour-initiating cells.

**Electronic supplementary material:**

The online version of this article (doi:10.1186/s12885-015-1394-7) contains supplementary material, which is available to authorized users.

## Background

Breast cancer, a neoplastic disease with high level of incidence and mortality, is the prevalent cancer in females [1, 2]. One reason for high rate of breast cancer, its metastatic potential and, in many cases, resistance to therapy, is the presence of tumour-initiating cells (TICs) [3, 4] that represent a small tumour subpopulation with the ability to self-renew and drive tumour growth [5, 6]. Recent research provides strong evidence for the contribution of TICs to tumour (re-) initiation and progression [7-12]. Therefore, specific therapies targeted at TICs may suppress tumour (re-) growth, perhaps even eliminating the pathology [13, 14]. Development of anti-TIC approaches is an emerging focus of research, and a group of compounds with anti-cancer properties acting by destabilising mitochondria, ‘mitocans’, appear to be efficient against TICs [15].

Mitocans define small compounds that induce apoptosis of malignant cells via targeting mitochondria. They are classified into several categories according to their molecular target [16]. Mitocans from the vitamin E (VE) group, epitomised by α-tocopheryl succinate (α-TOS), affect the mitochondrial complex II (CII) by interfering with the function of ubiquinone (UbQ), resulting in leakage of electrons and generation of reactive oxygen species (ROS), which trigger selective apoptosis in cancer cells [17, 18]. To promote its selective mitochondrial uptake driven by mitochondrial potential (ΔΨ_m,i_), we tagged α-TOS with the delocalised cation triphenylphosphonium (TPP^+^) to prepare mitochondrially targeted vitamin E succinate (MitoVES). This agent preferentially associates with mitochondria of cancer cells and kills malignant cells more efficiently than the parental compound [19, 20].

Selectivity of agents like MitoVES for malignant cells is based on the relatively high ΔΨ_m,i_ of cancer cells [21]. Recent reports document that TICs have higher ΔΨ_m,i_ than differentiated cancer cells [22]. Therefore we decided to establish a model of breast cancer TICs and test the anti-cancer efficacy of MitoVES.

## Methods

### Cell culture and sphere preparation

Breast cancer NeuTL cells derived from tumours of transgenic FVB/N *c-neu* mice [23] and human MCF7 cells obtained from the ATCC were cultured in DMEM with 10 % FBS and antibiotics. Spheres were prepared by seeding cells at the density of 10^5^/ml of ‘sphere medium’ composed of DMEM-F12 plus cell proliferation supplement (Neurocult), 10 ng/ml mouse or human recombinant EGF, 5 ng/ml recombinant FGF (R&D Systems), and 2 mM L-glutamine.

### Quantitative RT-PCR (qPCR)

Total RNA from cells or tissues was extracted using the RNeasy kit (Qiagen). The Revertaid First-Strand Synthesis System plus random hexamer primers (Thermo Fischer Scientific) were used to transcribe total RNA into cDNA. Using specific primers, genes of interest were evaluated with 2xSYBR Green (Qiagen) by means of the Eco qPCR System (Illumina). Target genes were normalised to *GAPDH*, and change in gene expression determined using the ΔΔCt method (see Additional file 1 for primer sequences).

### Cell cycle analysis

Adherent or sphere cells were fixed in 70 % ethanol overnight at -20 °C, pelleted and re-suspended in the staining solution (50 μg/ml propidium iodide, 100 μg/ml RNase A, 0.1 % Triton X-100). After 40 min incubation at 37 °C, samples were accessed with the Fortessa flow cytometer (BectonDickonson) and data analysed using the FlowJo software (TreeStar).

### Evaluation of mitochondrial membrane potential (ΔΨ_m,i_), reactive oxygen species (ROS), cell death and viability

Standard flow cytometric methods were applied utilising the following fluorescent probes. ΔΨ_m,i_ was estimated with tetramethylrhodamine methyl ester (TMRM), and ROS were evaluated using dichlorofluorescein diacetate (DCF) or MitoSOX. Apoptosis was evaluated using annexin V-FITC/propidium iodide. Viability was assessed using the MTT assay.

### Succinate dehydrogenase (SDH) and succinate quinone reductase (SQR) activity assays

For SDH activity, cells were seeded in 96 well plates at 10,000 cells per well and allowed to recuperate overnight. They were then incubated with 20 mM succinate for 1 h before 10 μl MTT reagent (5 mg/ml) was added to each well, followed by 4-h incubation at 37 °C and 5 % CO_2_. Media was then removed and formazan dissolved in DMSO, and absorbance was measured at 570 nm [19, 20]. For SQR activity, 40 μg of protein lysate extracted before the assay (Cell Lysis Buffer, Cell Signaling) were added to 1 ml of the SQR assay buffer (10 mM KH_2_PO_4_, pH 7.8, 2 mM EDTA, 1 mg/ml BSA, 80 μM DCPIP, 4 μM rotenone, 0.2 mM ATP and 10 mM succinate) and incubated at 30 °C for 10 min. Decylubiquinone was added to a final concentration of 80 μM, and absorbance assessed each minute for 30 min at 600 nm [19, 20].

### High-resolution respirometry

Oxygen consumption was assessed using the Oxygraph-2 k high-resolution respirometer (Oroboros). Intact cell respiration was evaluated with cells suspended in the RPMI medium without serum. Oxygen consumption was evaluated for cellular routine respiration, oligomycin-inhibited leak respiration, FCCP-stimulated uncoupled respiration (ETS) and rotenone/antimycin-inhibited residual respiration (ROX). Respiration via mitochondrial complexes was evaluated using saponin-permeabilised cells or shredded tumour tissue, suspended in the mitochondrial respiration medium MiR06. Oxygen consumption was evaluated for routine respiration, CI-linked respiration, (CI + CII)-linked respiration, maximum uncoupled respiration, CII-linked uncoupled respiration as well as residual oxygen consumption [24].

### Western blotting (WB)

Cells and homogenised tumour tissue were lysed, and total protein (30 μg) resolved by SDS-PAGE and transferred to PVDF membranes, which were probed with following antibodies: EpCAM, erbB2 (both from Sigma-Aldrich), caspase-9, caspase-8, cleaved caspase-3, VDAC, COX IV, SDHA (all from Cell Signaling), CD44, HSP60, actin (all from Abcam), CD133, PARP-1/2 (both from Santa Cruz), and SDHC (Novus Biologicals). ECL western blotting substrate (Thermo Scientific) and ChemiDoc™ XRS+ System (BioRad) were used to visualise and evaluate the blots.

### Native blue Gel eletrophoresis

Mitochondria were isolated following a standard protocol, and protein concentration assessed using the BCA assay. NativePAGE Novex Bis-Tris (4-16 % gradient) gels (Life Technologies) were used for electrophoresis of digitonin-solublised mitochondria. After electrophoresis, gels were incubated in the SDS-PAGE 1 × running buffer for 5 min, and the protein transferred to a PVDF membrane probed with specific antibodies against mitochondrial complex I (CI) (NUDFA9), CII (SDHA and SDHB), complex III (Core1), complex IV (COX Va) and complex V (ATPaseβ) (all antibodies from Cell Signaling). HSP60 was used as loading control.

### Preparation of SDHC knock-down cells

MCF7 cells were transfected with non-silencing (N.S.) or SDHC shRNA (both SABiosciences) using the FuGENE HD reagent as per standard protocol. Selected clones were tested for SDHC mRNA and protein, and the clone with lowest level of SDHC used in experiments.

### Tumour formation and MitoVES treatment

Tumours were established in female FVB/N *c-neu* mice (~2 months old) by subcutaneous grafting of NeuTL adherent or sphere cells at 3x10^6^ per animal. Mice were regularly checked by the Vevo770 ultrasound imaging (USI) apparatus equipped with a 30-μm resolution scan-head (VisualSonics). As soon as tumours reached ~50 mm^3^, animals were treated by intraperitoneal (i.p.) injection of MitoVES (25 nmol per gram of body weight) in corn oil containing 4 % ethanol every 3-4 d. Control mice were injected with the same volume (100 μl) of the excipient. Tumour progression was assessed by USI, which enables 3D reconstruction of tumours and precise quantification of their volume. Tumours were harvested, fixed in and paraffin-embedded. The blocks were cut into 1 μm sections stained with H&E or incubated with primary antibody and biotinylated secondary antibody. The ABC kit (Vector Laboratories) was used to amplify the signal. Mayer’s haematoxylin was used for counterstaining the nuclei. All animal experiments were performed according to the guidelines of the Australian and New Zealand Council for the Care and Use of Animals in Research and Teaching and were approved by the Griffith University Animal Ethics Committee.

### Statistical analysis

All data are mean values of at least three independent experiments ± S.D. The unpaired Student’s t test or one-way ANOVA were used to assess statistical significance. Differences with *p* < 0.05 were regarded as significant. Images are representative of three independent experiments.

## Results

### NeuTL and MCF7 spheres are enriched in TICs

**Fig. 1:**
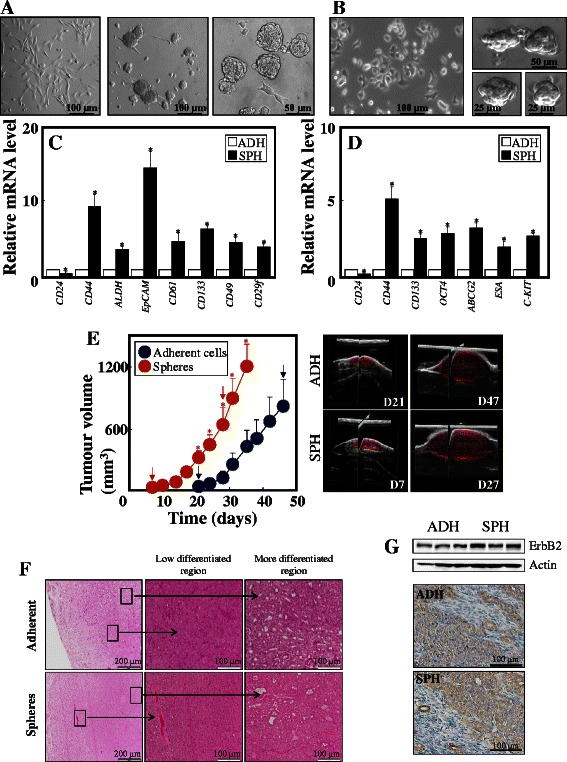
NeuTL and MCF7 spheres are a plausible model of TICs. Neu TL cells were cultured in serum-containing and sphere medium (**A**) and assessed for selected stemness genes by qPCR (**C**). MCF7 cells were cultured in adherent and ‘sphere’ medium (**B**) and assessed for selected stemness genes by qPCR (**D**). (**E**) NeuTL adherent and sphere cells were grafted s.c. in FVB/N c-neu mice (10^6^ cells per animal) and tumour volume assessed using USI. The images on the right are representative USI scans of tumours taken on the given days (indicated by arrows in the graph on the left). (**F**) Sections of tumours were stained by H&E for morphology, also showing regions of low and more differentiated cancer cells. (**G**) Tumour sections were evaluated for the level of erbB2 using WB and IHC. In all cases, the level of stemness genes in sphere cells was related to that in their adherent counterparts, set as 1. Data are mean values ± S.D. (n = 3). The symbol ‘*’ indicates statistically significant differences in the level of mRNA in adherent and sphere cells with p < 0.05. Images in panels A, B, E, F and G are representative of three independent experiments

To establish an *in vitro* model to study breast TICs, we grew NeuTL and MCF7 cells under condition that promotes sphere generation (Fig. 1 A, B). Both cell lines formed mammospheres within 3-5 days, reaching ~50 μm in diameter. To verify spheres as a model of breast TICs, mRNA level of a series of ‘stemness’ markers was assessed. As can be seen in Fig. 1 C, NeuTL spheres had higher expression of *CD44, ALDH*, *EpCAM*, *CD61*, *CD133*, *CD49* and *CD29f*, and lower expression of *CD24*, compared to their adherent counterparts. MCF7 spheres featured higher level of *CD44*, *CD133*, *OCT4*, *ABCG2*, *ESA* and *c-Kit*, and lower level of *CD24* (Fig. 1 D).

To assess their tumour-propagating efficacy, sphere and adherent cells were grafted into FVB/N *c-neu* mice. As shown in Fig. 1 E, NeuTL spheres initiated USI-detectable tumours within ~1 week, while there was a 2-week delay for adherent cells. Adherent cell-derived tumours progressed at about half the rate of the sphere-derived ones, with an increase by 100 mm^3^ in 1.4 and 2.7 days, respectively. Morphologically, the two types of tumours were similar, as documented by H&E staining (Fig. 1 F). As assessed by WB and IHC (Fig. 1 G), the receptor tyrosine kinase erbB2 was highly and similarly expressed in both tumour types.

### Breast TICs are resistant to chemotherapeutic drugs but sensitive to MitoVES

**Fig. 2:**
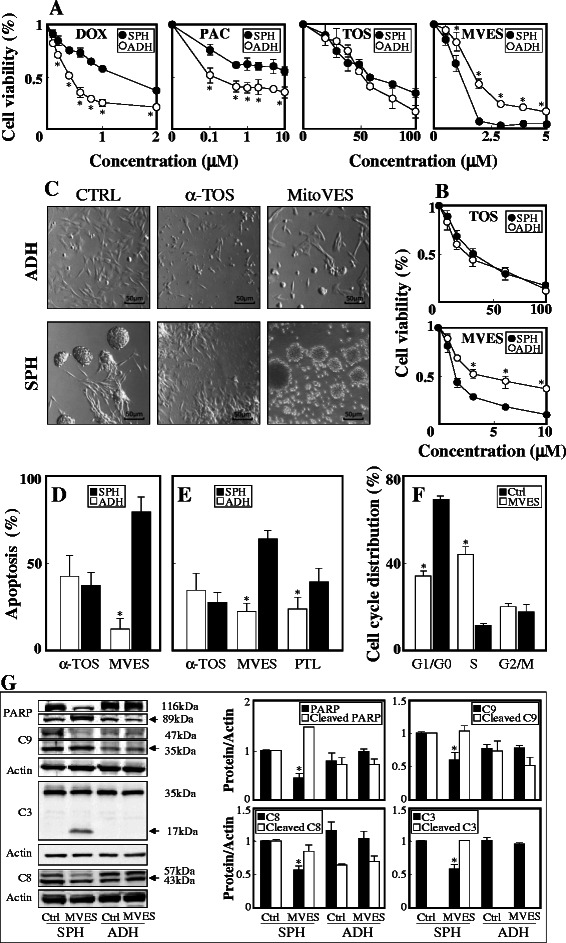
Breast TICs are resistant to chemotherapeutic drugs but sensitive to MitoVES. Adherent and sphere NeuTL (**A**) and MCF7 cells (**B**) were exposed to different concentrations of the agents for 24 h and viability assessed by the MTT assay. (**C**) NeuTL adherent and sphere cells were exposed to 50 μM α-TOS or 2 μM MitoVES for 24 h and inspected by light microscopy. Adherent or sphere NeuTL (**D**) or MCF7 cells (**E**) were exposed to α-TOS (50 μM), MitoVES (2 μM) or parthenolide (PTL; 10 μM) for 12 h and apoptosis evaluated using the annexin V/PI method. (**F**) Adherent NeuTL cells were exposed to 2 μM MitoVES for 24 h and evaluated for cell cycle distribution. (**G**) NeuTL sphere and adherent cells were exposed to 5 μM MitoVES for 12 h and full length and cleaved PARP, caspase-9 (C9), caspase-3 (C3) and caspase-8 assessed using WB with actin as loading control. The level of full length and cleaved proteins was evaluated by densitometry and related to actin. Data are mean values ± S.D. (n = 3). The symbol ‘*’ in panels A, B, D-F indicates statistically significant differences for adherent and sphere cells with p < 0.05. The symbol ‘*’ in panel G indicates statistically significant differences in the expression of the full length and cleaved protein with p < 0.05. Images in panel C are representative of three independent experiments

Figure 2 A documents that NeuTL spheres are more resistant to doxorubicin and paclitaxel compared to their adherent counterparts, consistent with their TIC nature. α-TOS killed adherent and sphere NeuTL and MCF7 cells with similar efficacy, while MitoVES was more efficient in killing sphere cells (Fig. 2 A, B). The IC_50_ values were higher for killing sphere cells by doxorubicin and paclitaxel, while they were significantly lower for MitoVES (Table 1). As the MTT assay used for cell viability partially relies on the oxidative capacity of mitochondria, the above results of α-TOS and MitoVES may be affected to some extent. Therefore further cell death assessment was carried on by flow cytometry using PI and Annexin IV staining. We can see that MitoVES also induced more cell death by apoptosis in sphere *vs*. adherent cells, while α-TOS was inefficient (Fig. 2 C-E). At 2 μM, MitoVES was more efficient in inducing apoptosis in MCF7 sphere cells that 10 μM parthenolide. While MitoVES at 2 μM was not very efficient in causing apoptosis in adherent NeuTL cells, it arrested their cell cycle (Fig. 2 F). The apoptotic nature of cell death induced in sphere cells by MitoVES is documented in Fig. 2 G. Apart from apoptotic proteins activated by MitoVES treatment, there were also certain amounts of cleaved Caspase-8 and cleaved Caspase-9 documented in the control group, which may be due to a small population of cells undergoing apoptosis among the whole cell culture.

**Table 1:** IC_50_ values (μM) for adherent and sphere cells exposed to various anti-cancer agents

Cell line	Doxorubicin		Paclitaxel		α-TOS		MitoVES	
	ADH	SPH	ADH	SPH	ADH	SPH	ADH	SPH
NeuTL	0.45 ± 0.07^a^	1.44 ± 0.17	0.26 ± 0.06	>10	53 ± 4.5	58 ± 6.3	1.9 ± 0.31	1.1 ± 0.25
MCF7	n.d.^b^	n.d.	n.d.	n.d.	24.8 ± 2.2	28.6 ± 1.9	4.52 ± 0.29	1.2 ± 0.25
MCF7 SDHC^low^	n.d.	n.d.	n.d.	n.d.	n.d.	n.d.	n.d.	8.2 ± 1.3

^a^IC_50_ values were calculated form the killing curves of the various adherent and sphere cell cultures exposed to the agents for 24 h. The killing curves were constructed using the MTT assay

^b^n.d.: not determined

### Increased killing of breast TICs by MitoVES involves mitochondria

**Fig. 3:**
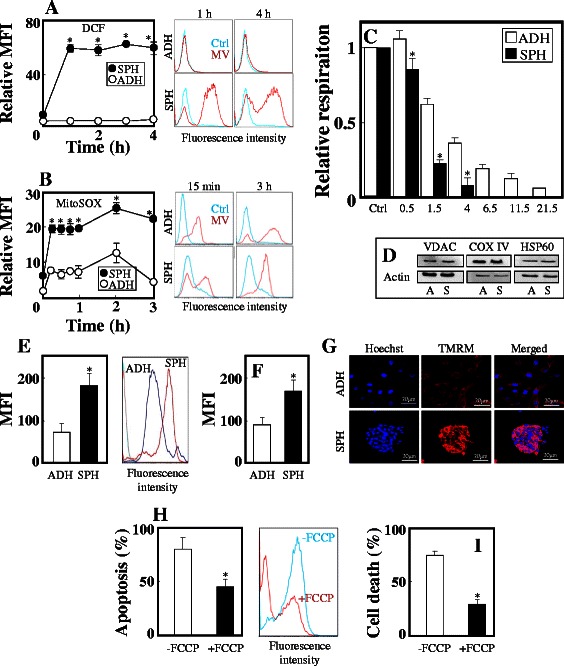
Mitochondria play a role in high TIC killing activity of MitoVES. NeuTL adherent and sphere cells were exposed to 2 μM MitoVES for the times shown and ROS evaluated by flow cytometry using DCF (**A**) or MitoSOX (**B**), and expressed as relative mean fluorescence intensity (MFI). The histograms on the right are representative of individual readings. (**C**) Adherent and sphere NeuTL cells were assessed for routine respiration in the absence or presence of MitoVES at the concentrations shown (μM). (**D**) Adherent and sphere NeuTL cells were probed by WB for the levels of mitochondrial markers with actin as loading control. Adherent and sphere NeuTL (**E**) and MCF7 cells (**F**) were evaluated for ΔΨ_m,i_ using TMRM and flow cytometry. The histogram in panel E on the right is an example of a reading for NeuTL cells. (**G**) Adherent and sphere NeuTL cells were labelled with Hoechst to visualise nuclei and TMRM to document ΔΨ_m,i_, and inspected by confocal microscopy. Sphere NeuTL (**H**) and MCF7 (**I**) cells were exposed to 2 μM MitoVES for 24 h in the absence or presence of 10 μM FCCP and apoptosis evaluated. The histogram in panel H on the right is an example of reading for NeuTL cells. Data are mean values ± S.D. (n = 3). The symbol ‘*’ in panels A-C, E and F indicates statistically significant differences for adherent and sphere cells with p < 0.05. The symbol ‘*’ in panels H and I indicates statistically significant differences in apoptosis induced in the presence and absence of FCCP with p < 0.05. Images in panels C and D are representative of three independent experiments

MitoVES was more efficient in ROS generation in sphere than adherent cells, in particular when assessed with DCF (Fig. 3 A, B). MitoVES also more efficiently suppressed respiration in sphere compared to adherent cells (Fig. 3 C). That the two types of cells do not differ in mitochondrial mass was confirmed by WB (Fig. 3 D). Both NeuTL and MCF7 spheres showed considerably higher ΔΨ_m,i_ potential than their adherent conterparts (Fig. 3 E-G). Important role of ΔΨ_m,i_ in apoptosis induction by MitoVES follows from an experiment, in which the mitochondrial uncoupler FCCP inhibited MitoVES-induced killing in NeuTL and MCF7 spheres (Fig. 3 H, I). The higher ΔΨ_m,i_ in sphere cells may enrich more MitoVES into their mitochondrial, which contribute to the high susceptibility of spheres upon MitoVES treatment in comparison with their adherent counterparts. Moreover, it is also found that NeuTL sphere cells have higher expression of mitochondrial complexes (unpublished data), some of which function as the molecular targets of MitoVES.

### MitoVES affects mitochondrial complexes of breast TICs

**Fig. 4:**
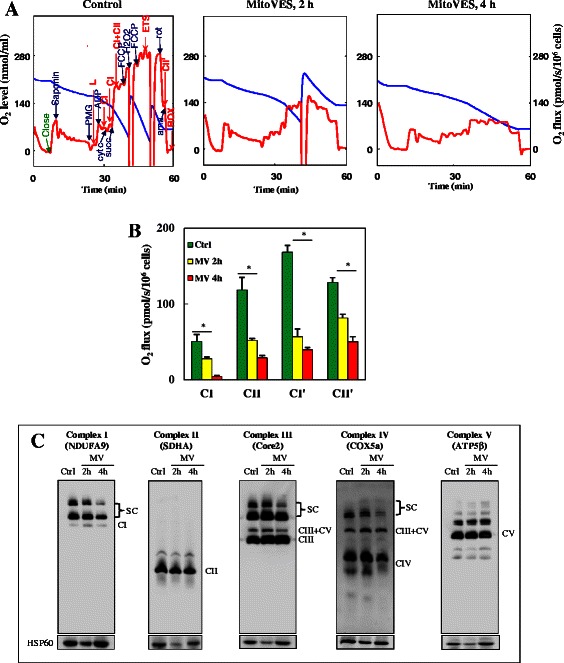
MitoVES affects mitochondrial complexes. (**A**) NeuTL spheres were treated with 2 μM MitoVE for and 4 h, before they were harvested, permeablised with saponin and evaluated for respiration at the presence of substrates specific for CI and CII using the protocal indicated in more detail in Materials and Methods. The abbreviations in the top left line graph are: L, leak; CI, complex I; CII, complex II; ETS, electron transfer system (uncoupled resiraiton); CII’, uncoupled respiration via CII; ROX, residual respiration; PMG, pyruvate, malate and glutamate; cyt c, cytochrome c; succ, succinate; F, FCCP; rot, rotenone; ama, antimycin A. (**B**) The respiration via CI and CII, and the uncoupled respiration via CI (CI’) and CII (CII’) as derived from results shown in panel A is documented in control cells and cells exposed to 10 μM MitoVES for 2 and 4 h. (**C**) The mitochondrial fraction, prepared from control NeuTL cells or cells exposed to 10 μM MitoVES for 2 and 4 h, was lysed in the presence of digitonin and subjected to native blue gel electrophoresis as detailed in Materials and Methods. Specific subunits of individual complexes were detected using the antibodies as shown. HSP60 was used as a loading control. The symbol ‘*’ in panels indicates statistically significant differences (p < 0.05) for the respiration after cells were exposed to MitoVES

We tested the contribution of CI and CII to respiration of breast cancer cells and whether this is affected by MitoVES. As shown in Fig. 4 A & B, oxygen consumption was inhibited more at the level of CII, the target of the agent. This was observed for both coupled and uncoupled state of respiration. Native blue gel electrophoresis using a mild detergent followed by WB was employed to assess the change of mitochondrial respiratory complexes and supercomplexes upon MitoVES treatment. Some decrease in the level of supercomplexes in cells treated with MitoVES was observed after 2 and 4 h of exposure to the drug (Fig. 4 C).

### MitoVES efficiently suppresses tumour growth

**Fig. 5:**
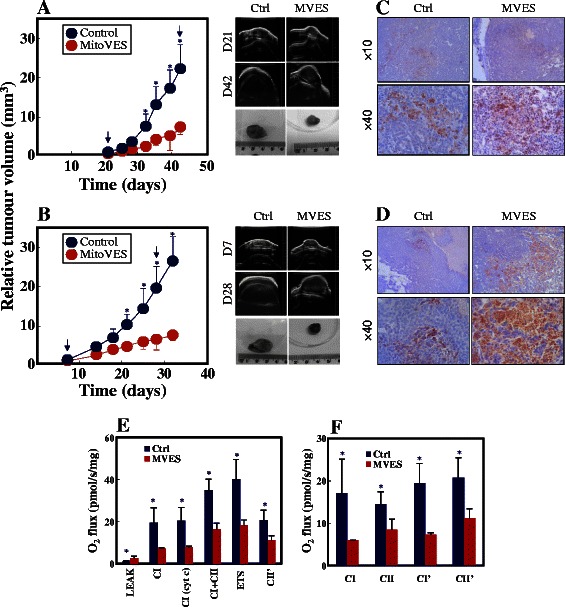
MitoVES suppresses tumour progression. NeuTL adherent (**A**) and sphere cells (**B**) were grafted s.c. in FVB/N c-neu mice (10^6^ cells per animal) and tumour volume assessed in control and MitoVES-treated animals using USI. The images on the right are representative USI scans of tumours taken on the given days (indicated by arrows in the graph on the left), the images on the right also show representative tumours excised from mice at the end of the experiment. Tumours derived from adherent (**C**) and sphere NeuTL cells (**D**) were paraffin-embedded, sectioned and probed by IHC for cleaved caspase-3. Tumour tissue was shredded and oxygen consumption evaluated using oxygraph. The respiration via mitochondrial complexes was assessed and calculated (**E, F**). Data are mean values ± S.D. (n = 3). The symbol ‘*’ in panels A and B indicates statistically significant differences in the volume of control and MitoVES-treated tumours with p < 0.05. Images in panels C and D are representative of three independent experiments. The symbol ‘*’ in panel F and G indicates statistically significant differences in the respiration levels of control and MitoVES-treated tumours with p < 0.05

Adherent and sphere NeuTL cells were subcutaneously injected in FVB/N *c-neu* mice to form syngeneic tumours, after which MitoVES was administrated. As revealed by USI, MitoVES efficiently suppressed growth of tumours derived from both types of cells, such that after 7-8 injections, the tumour volume was lower by ~80 % in the treated vs. control mice (Fig. 5 A, B). MitoVES suppressed tumour growth by way of inducing apoptosis, as documented by IHC using an antibody to cleaved caspase-3 (Fig. 5 C, D). Assessment of respiration revealed that MitoVES suppressed both CI- and CII-dependent respiration of tumours (Fig. 5 E, F).

### MitoVES kills breast TICs in a complex II-dependent manner

**Fig. 6:**
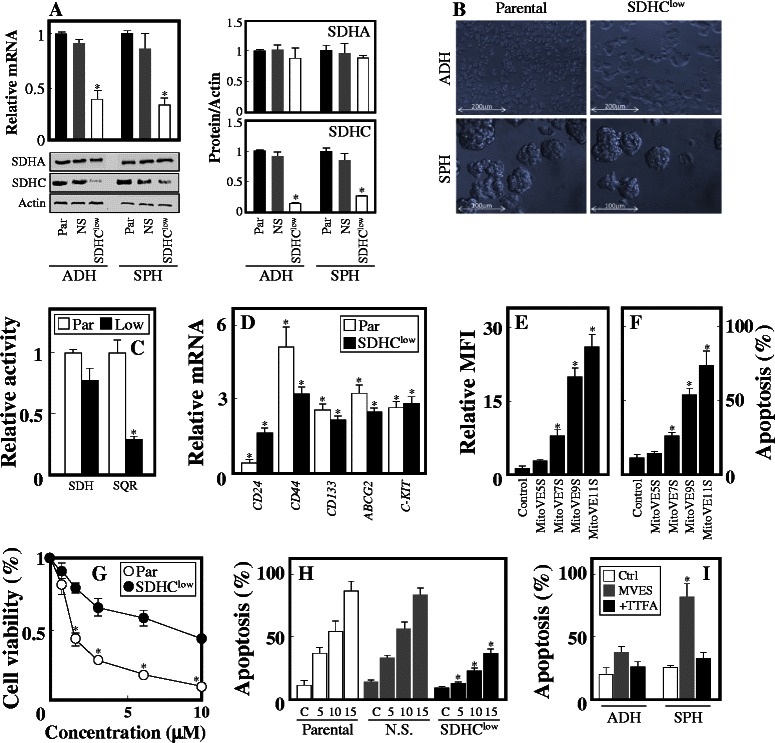
Apoptosis induced by MitoVES is dependent on complex II. (**A**) Adherent and sphere MCF7 cells were transfected with non-silencing (NS) and SDHC shRNA and assessed for the level of SDHA and SDHC by qPCR and WB with actin as loading control. The graphs on the right show the level of the SDHA and SDHC proteins in the sub-lines related to actin. (**B**) Parental and SDHC^low^ MCF7 cells were grown in serum-containing and ‘sphere’ medium and inspected by light microscopy. Parental and SDHC^low^ MCF7 sphere cells were evaluated for SDH and SQR activities (**C**) and for the level of stemness genes related to their level in MCF7 adherent cells set as 1 (**D**). MCF7 sphere cells were exposed to MitoVES homologues at 5 μM for 1 h and assessed for ROS using MitoSOX (**E**) and for 12 h and assessed for apoptosis (**F**). Parental, NS and SDHC^low^ MCF7 sphere cells, as shown, were exposed to MitoVES for 24 h (viability) or 12 h (apoptosis) and evaluated for viability using the MTT assay (**G**) and apoptosis by the annexin V/PI method (**H**). (**I**) Adherent and sphere MCF7 cells were evaluated for apoptosis after 24 h exposure to 5 μM MitoVES in the absence or presence of 10 μM TTFA. Data are mean values ± S.D. (n = 3). The symbol ‘*’ in panels A-C indicates statistically significant differences for parental and SDHC^low^ MCF7 cells, in panel D for adherent and sphere cells, in panels E and F for control and treated cells, in panels G and H for parental and SDHC^low^ MCF7 cells, and in panel I for cells treated in the absence and presence of TTFA, with p < 0.05. Images in panel B are representative of three independent experiments

Whether MitoVES induces apoptosis in breast TICs via CII has not been tested. We therefore knocked down the SDHC subunit of CII in MCF7 cells and found that SDHC^low^ MCF7 cells form spheres with low level of SDHC, while SDHA is unaffected (Fig. 6 B). SDH activity of CII, residing in SDHA, was only marginally affected, while SQR activity of CII that requires intact SDHC was suppressed (Fig. 6C). SDHC^low^ MCF7 spheres feature high level of stemness, as documented by several TIC markers (Fig. 6D). Treatment of MCF7 sphere cells with MitoVES homologues differing in the length of the aliphatic chain linking the tocopheryl succinyl group TPP^+^ group revealed that the short-chain homologues are inefficient in ROS generation and apoptosis induction (Fig. 6 E, F), pointing to CII as a target. SDHC^low^ MCF7 spheres showed higher viability in the presence of MitoVES than MCF7 spheres (Fig. 6G) with the IC_50_ value ~4-fold higher (Table 1). SDHC^low^ spheres were also more resistant to MitoVES-induced apoptosis than their parental counterparts (Fig. 6H). Finally, thenoyltrifluoroacetate (TTFA), an agent binding to CII’s UbQ site, prevented apoptosis induced by MitoVES.

## Discussion

In this communication we describe a sphere model of breast TICs that was adapted from research on neural stem cells isolated from the CNS [25, 26] and that is now accepted as a model for TIC studies in tissue culture [27-29]. The increased level of stemness in NeuTL and MCF7 spheres documented by expression of specific markers [7, 30-34] is consistent with our recent results using microarray chip approach [35]. Additional evidence for the plausibility of spheres as a TIC model is documented by their higher tumour-initiating/propagating efficacy [7]. Furthermore, the ‘sphere’ TICs were found resistant to established, first line breast cancer therapeutics, which more efficiently killed adherent breast cancer cells, in line with the notion of general recalcitrant nature of TICs [12, 36, 37].

However, we found that breast TICs are killed more efficiently by the mitocan MitoVES that by pathenolide, an agent that was shown to cause death of TICs [38, 39]. MitoVES accumulates in mitochondria on the basis of high ΔΨ_m,i_ due to the presence of the TPP^+^ group [10, 11, 40], and this is consistent with the notion of higher ΔΨ_m,i_ in stem cells [41]. MitoVES inhibits the respiration of breast TICs via mitochondrial CI and, even more, CII, causing the generation of ROS, which leads to apoptosis of these cells. The assembly of mitochondrial supercomplexes was inhibited, to some extent, as well. MitoVES also suppressed progression of tumours derived from both adherent and sphere cells, with similar efficacy. The likely reason for this is that, when grafted, TICs differentiate within the tumour microenvironment into fast-proliferating tumour cells [28]. In tumour tissue, inhibition of cell respiration and induction of apoptosis were also documented. Using the syngeneic FVB/N c-neu mouse model, the drug effect against erbB2^high^ breast tumour was investigated under conditions of functional immune system.

Of interest is the mechanism by which MitoVES kills breast TICs. Our previous data document that the mitochondrially targeted agent, similarly as the untargeted α-TOS, acts via interacting with the UbQ site of CII [17-20]. That MitoVES acts also by targeting the UbQ site in CII of breast TICs was first indicated by experiments, in which shorter homologues of full length MitoVES (11 carbons in the aliphatic chain linking the tocopheryl succinyl and TPP^+^ groups) were correspondingly less efficient in ROS generation and apoptosis induction. Our recent molecular modelling indicates that this linker has to be of certain length so that the biologically active moiety of MitoVES can reach the UbQ site of CII buried in the inner mitochondrial membrane; the reason being that the TPP^+^ group anchors the positively charged end of the molecule at the matrix face of the inner mitochondrial membrane [19, 20]. Importantly, spheres derived from MCF7 cells with knocked down SDHC, lacking the UbQ site, were resistant to MitoVES treatment. Further, we show that the presence of TTFA, a small molecule that is known to bind to CII’s UbQ site [42], prevented the killing activity of MitoVES in sphere cells. Collectively, our data convincingly document that MitoVES targets the UbQ site in CII to efficiently kill breast TICs, and are consistent with the notion of CII as an intriguing, novel target for anti-cancer agents [43]. Importantly, subunits of CII only rarely mutate, such that their mutational frequency is high in neoplasias like familial paraganglioma, but only one in one million breast cancer patients features a CII mutation [44].

Thus, we document a high killing activity of MitoVES towards breast cancer TICs that are resistant to several established anti-cancer agents. While our findings are of translational significance, we also, for the first time, document a link between mitochondrial complex II and killing of tumour-initiating cells. The combination therapy of MitoVES which effectively kills breast TICs and established anti-cancer drugs targeting highly proliferating cells may lead to a better tumour suppression results, which will be further investigated in our future research.

## Conclusions

In this project, mammosphere models for studying breast TICs were established and verified. These cells featured altered mitochondrial function. A mitochondrially targeted anti-cancer compound, mitocan, epitomised by mitochondrially targeted vitamin E succinate (MitoVES), was found very efficient in killing TICs, which has a potential translational relevance. Additional studies are needed to explore the clinical value of our study for using MitoVES as an agent that can potentially eradicate cancer stem-like cells alone or in combination with other anti-cancer drugs.

## Additional file

Additional file 1:
**Primers used for qPCR analyses.**

## Abbreviations

ADH: Adherent
CI: Complex I
CII: Complex II
DCF: Dichlorofluorescein diacetate
H & E: Haematoxylin & eosin
IHC: Immunohistochemistry
MitoVES: Mitochondrially targeted vitamin E succinate
PI: Propidium iodide
ROS: Reactive oxygen species
SPH: Sphere
TICs: Tumour-initiating cells
α-TOS: α-tocopheryl succinate
TMRM: Tetramethylrhodamine methyl ester
TPP^+^: Triphenylphosphonium
TTFA: Thenoyltrifluoroacetate
UbQ: Ubiquinone
USI: Ultrasound imaging
