# Supplementary material for: Mitochondrially targeted vitamin E succinate efficiently kills breast tumour-initiating cells in a complex II-dependent manner
Source: BMC Cancer. 2015 May 13;15:401. doi: 10.1186/s12885-015-1394-7 (PMC4494715; doi:10.1186/s12885-015-1394-7)
Supplement: Additional file 1: — Primers used for qPCR analyses. [file 12885_2015_1394_MOESM1_ESM.doc]

# Supplementary Material

Primers used for qPCR analyses

| **Gene** | **Forward** | | | | **Reverse** |
| --- | --- | --- | --- | --- | --- |
| **Mouse genes** | | | | | |
| *CD24* | | GCG GAC ATG GGC AGA GCG ATG | | GCG TGG GTA GGA GCA GTG CCA | |
| *CD44* | | CGG AGC ACC TTG GCC ACC ATT | | TGC ACT CGT TGT GGG CTC CTG A | |
| *ALDH* | | TTG CGT CCA CTC TGA GGG CAA | | CAC GGG CCC GGT TCA CGA TG | |
| *CD29* | | AAC TTG TTG GTC AGC AAC GC | | AGC CAA TCA GCG ATC CAC AA | |
| *CD49f* | | GCA CCT CGG GCA GAA GCA CT | | CCG CGA CTC CTG CTT CGT GTT | |
| *CD61* | | ACG AGC CAG AGC CAA GTG GGA | | CCC ACG GTC CTG GCG TCA TC | |
| *CD133* | | CCT CAA CGT GGT CCA GCC GAA | | GCA GCC CAC CAG AGG CAT GA | |
| *EpCAM* | | CTG GCG TCT AAA TGC TTG GC | | CCT GTT CGG TTC TTC GGA CTC | |
| **Human genes** | | | | | |
| *CD24* | | AGC GGT TCT CCA AGC ACC CA | TAG GAG CAG TGC CAG CAG CA | | |
| *CD44* | | ACC TGG GAT TGG TTT TCA TGG TTG T | TTC ATT TGG CTC CCA GCC TGC | | |
| *CD133* | | CCC TAA ATT TGC ATG AAA GCA CAA GGT | CAA CGT TAA ATT TTG TCC GAC CAG TTC | | |
| *OCT4* | | GGA GGC CCC ATC GGA GTT GC | CCC CAG GTT GGA GTG GGG CT | | |
| *ABCG2* | | CTG AGA TCC TGA GCC TTT GG | TGC CCA TCA CAA CAT CAT CT | | |
| *ESA* | | CGC AGC TCA GGA AGA ATG TG | TGA AGT ACA CTG GCA TTG ACG | | |
| *C-KIT* | | TGA TGC CTT CAA GGA CTT TGT | TTT TTG GCC TTC CCT TTC TC | | |
| *SDHA* | | AAG AAG GAG TAC ATT GAA GGG AGA T | TTT GGA AAT AAA AGA AAG TTT GTT A | | |
| *SDHC* | | CATTAT TAC ATT CAC CCA TCT TTC TG | AGA CCC TTT TCC ACT ATT ATG GTC TA | | |
